# Case Report: Zinc deficiency as a modifiable risk factor for enfortumab vedotin–induced cutaneous adverse events

**DOI:** 10.3389/fonc.2025.1690770

**Published:** 2025-11-28

**Authors:** Aya Nishizawa, Masaya Itoh, Midori Inoue, Moe Makiguchi, Fumitaka Koga

**Affiliations:** 1 Department of Dermatology, Tokyo Metropolitan Cancer and Infectious Diseases Center, Komagome Hospital, Tokyo, Japan; 2 Department of Urology, Tokyo Metropolitan Cancer and Infectious Diseases Center, Komagome Hospital, Tokyo, Japan

**Keywords:** enfortumab vedotin, cutaneous adverse events, zinc deficiency, SDRIFE-like rash, zinc supplementation

## Abstract

**Background:**

Enfortumab vedotin (EV) is effective for advanced urothelial carcinoma, but cutaneous adverse events (CAEs) remain a major barrier to treatment continuity. Intertriginous eruptions resembling symmetrical drug-related intertriginous and flexural exanthema (SDRIFE) are particularly problematic. Zinc deficiency causes similar dermatoses, suggesting a possible link with EV-induced skin toxicity.

**Case presentation:**

We retrospectively analyzed 10 EV-treated patients with advanced urothelial carcinoma. Serum zinc levels were measured at the onset of CAEs or dysgeusia, and all patients had subnormal zinc levels (80–130 μg/dL). SDRIFE-like rash occurred in six patients, dry skin in five, and maculopapular rash in four. Patients with SDRIFE-like rash had significantly lower zinc levels than those without (median: 63 μg/dL vs. 70 μg/dL, P = 0.041). Zinc supplementation (100–150 mg/day, 2–4 weeks) was administered in four patients with grade ≥2 skin rash and dysgeusia. Three with SDRIFE-like rash improved within days and resolved within 14 days despite poor response to corticosteroids, whereas dry skin improved only partially and dysgeusia did not improve.

**Discussion:**

These findings suggest that zinc deficiency may predispose EV-treated patients to SDRIFE-like eruptions by amplifying skin vulnerability typical of zinc-deficient states. Zinc supplementation showed rapid benefit in corticosteroid-refractory cases, pointing to its potential as a supportive adjunct.

**Conclusion:**

Zinc deficiency may represent a modifiable factor in EV-induced skin toxicity, particularly SDRIFE-like rash. Monitoring zinc status and considering supplementation could help mitigate rash severity and support treatment adherence and continuity, which is critical for patients with advanced urothelial carcinoma receiving EV as one of the last available treatment options.

## Introduction

Enfortumab vedotin (EV), an antibody–drug conjugate targeting nectin-4, is an established treatment for advanced urothelial carcinoma (UC), particularly following immune checkpoint inhibitors (1). However, cutaneous adverse events (CAEs) occur in approximately 47% of patients treated with EV (2). These toxicities frequently affect intertriginous, flexural, and acral regions and may require treatment interruption or dose modification (3).

Among EV-related CAEs, symmetrical drug-related intertriginous and flexural exanthema (SDRIFE)-like rashes occur with relatively high frequency and warrant particular attention, as they can lead to treatment interruption or discontinuation (4). Notably, these rashes resemble zinc deficiency–related dermatoses, such as acrodermatitis enteropathica, which characteristically affect periorificial and intertriginous areas (5). Moreover, zinc deficiency is associated with systemic manifestations—including dry skin, dysgeusia, and diarrhea—many of which overlap with EV-related toxicities (6).

Zinc deficiency in patients with UC may result from chronic inflammation, impaired nutritional status, and cancer-related metabolic alterations (7–9). In addition, previous studies have shown that anticancer agents such as EGFR inhibitors and tyrosine kinase inhibitors can reduce serum zinc levels and aggravate dermatologic toxicity (10, 11). Whether EV exerts similar effects on zinc homeostasis, thereby contributing to skin toxicity, remains unclear.

## Case description

Between January and August 2024, 14 patients received EV therapy for immune checkpoint inhibitor–refractory advanced UC at our institution. Among these, 10 patients in whom serum zinc levels were assessed during the treatment course were included in this report.

Serum zinc levels were measured at the onset of clinical features suggestive of zinc deficiency (e.g., cutaneous eruptions, dysgeusia, diarrhea), with repeat assessments performed as clinically indicated.

In selected patients, zinc supplementation was introduced using zinc acetate hydrate (100–150 mg/day for 2–4 weeks). Following supplementation, topical treatment was adjusted: corticosteroid ointments were discontinued and replaced with either white petrolatum or heparinoid-based moisturizers.

Clinical data were retrospectively retrieved from medical records, including serum zinc levels, prior treatments before EV, duration of EV therapy, presence and type of CAEs, time to CAE onset, and occurrence of dysgeusia. In patients who received zinc supplementation, changes in both clinical symptoms and serum zinc levels were documented to evaluate treatment response.

This retrospective case series was approved by the institutional review board of Tokyo Metropolitan Cancer and Infectious Diseases Center, Komagome Hospital (IRB #3320).

## Results

This study included 10 male patients with a median age of 75 years (range: 61–80 years). All patients had received prior systemic therapy before initiating EV, consisting of platinum-based chemotherapy (gemcitabine plus cisplatin or carboplatin) and/or immune checkpoint inhibitors, including adjuvant nivolumab (n = 1), switch-maintenance avelumab (n = 6), and pembrolizumab (n = 5). (**Table 1**).

**Table 1:** EV-related cutaneous adverse events and serum zinc levels.

Patient characteristics					
Age, median (range), years			75 (61-80)		
Male sex, n (%)			10 (100%)		
			Number of patients		
Systemic therapy prior to EV therapy (n=10)	GC		8		
	G-CBDCA		5		
	Avelumab		6		
	Pembrolizumab		5		
	Nivolumab		1		
Line of systemic therapy for EV (n=10)	3rd line		2		
	4th line		8		
EV therapy (n=10)	Cycle, median (range)		8 (2-14)		
	Duration, median (range), wks		32 (8-48)		
Cutaneous adverse events (CAEs) (n=8)					
Onset from the first EV infusion to CAEs onset, median (range), days			23 (15-50)		
CTCAE grade of CAEs					
Grade		Number of patients	Types of CAEs^※^ (n)		
All Grade		8			
Grade 1		2	Dry skin (2), maculopapular rash (2)		
Grade 2		4	Dry skin (3), SDRIFE like rash (4), maculopapular rash (2)		
Grade 3		2	SDRIFE like rash (2)		
Adverse events			Serum zinc level, median (range), µg/dL		*p* value^#^
			Yes	No	
Types of CAEs (n=8), duplicate entries	All types (n=8)		65 (38-79)	70 (64-79)	0.20
	Dry skin (n=5)		58 (38-79)	69 (39-79)	0.25
SDRIFE-like rash (n=6)		62 (38-72)	70 (56-79)	0.041
Maculopapular rash (n=4)		68 (56-72)	64 (38-79)	0.42
Zinc supplementation effects(n=4)			Number of patients		
yes	no	
CAEs	SDRIFE-like rash		3	0	
	Dry skin		0	2	
Dysgeusia			0	4	

Summary of patient characteristics, prior therapies, EV treatment details, types and severity of cutaneous adverse events (CAEs), and their association with serum zinc levels. Effects of zinc supplementation are also included.

#P value calculated using the Mann–Whitney U test; ※Duplicate entries included.

EV, enfortumab vedotin; GC, gemcitabine plus cisplatin; G-CBDCA, gemcitabine plus carboplatin; CAEs, cutaneous adverse events; SDRIFE, symmetrical drug-related intertriginous and flexural exanthema; CTCAE, Common Terminology Criteria for Adverse Events, version 5.0.

Cutaneous adverse events (CAEs) occurred in eight patients. The most frequent manifestation was SDRIFE-like rash (n = 6), followed by dry skin (n = 5) and maculopapular rash (n = 4). The median time to initial CAE onset after the first EV infusion was 23 days (range: 15–50 days). CAEs were graded as 3 in two patients, 2 in four patients, and 1 in two patients. Both patients with grade 3 events required treatment interruption and dose reduction; however, none permanently discontinued EV due to CAEs. Other EV-related adverse events included dysgeusia (n = 7), peripheral neuropathy, diarrhea, fatigue, and weight loss.

Serum zinc levels were measured at the onset of rash or dysgeusia, and all 10 patients had subnormal values below the normal range (80–130 μg/dL). The median timing of zinc measurement was 144 days after the first EV dose (range: 15–337 days). In five patients with serial assessments, zinc levels continued to decline by 5–10 µg/dL within 4–8 weeks in four who continued EV, whereas one patient who discontinued EV due to CAEs showed an increase of >10 µg/dL after one month.

We compared serum zinc levels according to the presence or absence of each CAE type (dry skin, SDRIFE-like rash, and maculopapular rash). Notably, only SDRIFE-like rash was significantly associated with lower zinc levels, with median values of 63 µg/dL in patients with the rash versus 70 µg/dL in those without (P = 0.041; **Table 1**).

**Figure 1:**
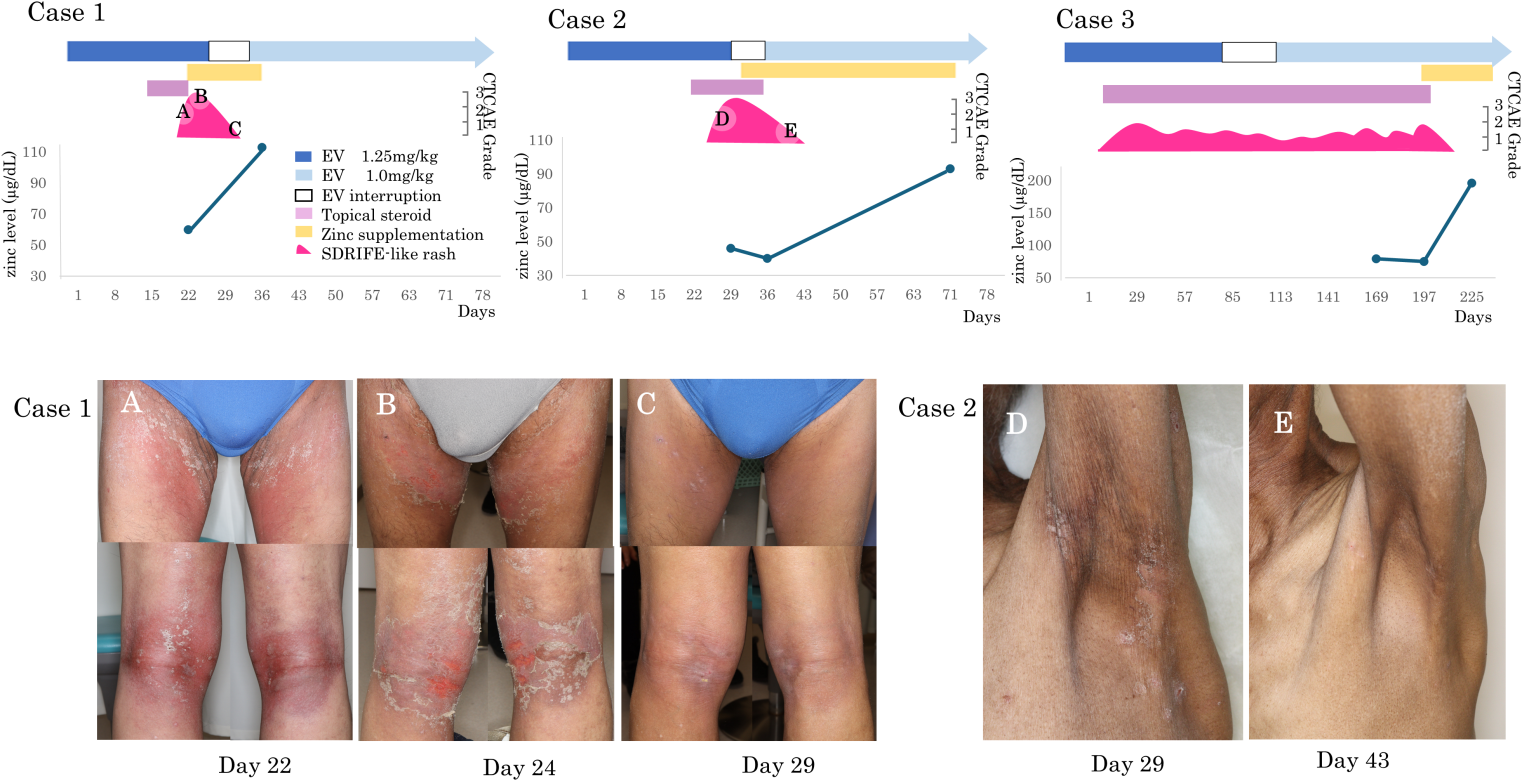
SDRIFE-like rash and serum zinc levels during EV therapy. Clinical timelines and representative skin findings in two patients with EV-induced SDRIFE-like rash who received zinc supplementation. Both patients had subnormal serum zinc levels and were refractory to topical corticosteroids. Marked improvement was observed after zinc supplementation and transition to emollient therapy. **(A)** Case 1, Day 22: Erythematous scaling rash in intertriginous areas. **(B)** Case 1, Day 24: Progression to painful erosions despite topical corticosteroid use. **(C)** Case 1, Day 29: Improvement after zinc supplementation and switch to moisturizers. **(D)** Case 2, Day 29: SDRIFE-like erythema and scaling with dryness, persistent under corticosteroid therapy. **(E)** Case 2, Day 43: Marked improvement of SDRIFE-like rash and partial resolution of dryness after two weeks of zinc supplementation with moisturizers alone.

Zinc supplementation (100–150 mg/day for 2–4 weeks) was administered in four patients with grade ≥2 skin rash and dysgeusia. Among them, two had SDRIFE-like rash alone, one had SDRIFE-like rash with dry skin, and one had dry skin alone (**Table 2**). The three patients with SDRIFE-like rash showed improvement within a few days and resolution within 14 days, even though they had responded poorly to corticosteroids (**Figure 1**). In contrast, dry skin showed partial improvement but did not completely resolve, and dysgeusia remained unchanged. Serum zinc levels normalized in all four patients within 1–4 weeks, and none required permanent EV discontinuation. These findings suggest that zinc deficiency may contribute to the development of SDRIFE-like rash during EV therapy.

**Table 2:** Clinical response to zinc supplementation in four EV-treated patients.

Case	CAE type(s)	Cutaneous CTCAE grade	Response to zinc supplementation
1	SDRIFE-like rash, dysgeusia	3	Rash improved; dysgeusia unchanged
2	SDRIFE-like rash and dry skin, dysgeusia	3	Rash improved; dry skin partially improved; dysgeusia unchanged
3	SDRIFE-like rash, dysgeusia	2	Rash improved; dysgeusia unchanged
4	Dry skin, dysgeusia	2	Dry skin partially improved; dysgeusia unchanged

Response to zinc supplementation is described as improvement of SDRIFE-like rash unless otherwise specified.

CTCAE, Common Terminology Criteria for Adverse Events; CAE, cutaneous adverse event; SDRIFE, symmetrical drug-related intertriginous and flexural exanthema; EV, enfortumab vedotin.

## Discussion

Zinc deficiency can arise from multiple causes, including inadequate dietary intake, gastrointestinal disorders, renal or hepatic dysfunction, chronic infections, and malignancies such as urothelial carcinoma (UC) (6–8). Anticancer therapies have also been implicated in zinc depletion. In particular, epidermal growth factor receptor (EGFR) inhibitors and tyrosine kinase inhibitors (TKIs) have been reported to lower serum zinc levels, potentially contributing to dermatologic toxicities such as xerosis and pruritus (10, 11). Although the precise mechanisms remain unclear, these findings support the notion that some targeted agents may interfere with zinc homeostasis.

In our cohort, all patients exhibited subnormal serum zinc levels during EV therapy. Moreover, patients who continued EV after the onset of skin toxicity or dysgeusia showed further reductions in zinc levels, whereas in one patient who discontinued EV, serum zinc increased from baseline at one month after discontinuation. These observations raise the possibility that EV itself may exacerbate zinc depletion, potentially through off-target effects on epithelial homeostasis given the abundant expression of nectin-4 in the skin (1–3). Moreover, nectin-4 plays a key role in cell–cell adhesion and epithelial barrier integrity; its inhibition may indirectly alter zinc transport or utilization in keratinocytes. Disruption of epithelial junctions could further increase transepidermal water and micronutrient loss, thereby aggravating pre-existing zinc deficiency (12, 13).

Zinc plays a critical role in skin integrity, keratinocyte differentiation, and wound healing (9). In acrodermatitis enteropathica—a prototypical zinc deficiency dermatosis—sharply demarcated erythema frequently occurs in intertriginous and periorificial regions, accompanied by vesicles, erosions, and crusting.^5^ The SDRIFE-like rash observed in EV-treated patients resembles this condition both anatomically and morphologically.³,^4^ In our study, patients with SDRIFE-like rash had significantly lower serum zinc levels than those without, and zinc supplementation led to rapid improvement of the rash in all treated cases. Similar benefit has been reported in skin toxicities induced by EGFR inhibitors (11), supporting a role for zinc supplementation in therapy-associated rashes.

In contrast, dry skin showed only partial improvement, and dysgeusia did not consistently respond to supplementation. These differences suggest that distinct pathophysiological mechanisms may underlie each symptom, potentially involving cumulative epithelial damage from prior therapies, EV-specific off-target effects unrelated to zinc status, or more complex alterations in skin barrier or taste receptor function.

The three patients who received zinc supplementation for grade ≥2 skin rash were able to continue EV therapy without further dose reductions. In advanced UC, maintaining therapeutic intensity with EV is critical for disease control and survival. Thus, addressing potentially modifiable factors, such as zinc deficiency, may have important clinical implications.

This study has several limitations. First, its retrospective design and small sample size limit the generalizability of the findings and should be interpreted as hypothesis-generating. Second, serum zinc levels before EV initiation were not available, preventing definitive conclusions regarding causality or the temporal relationship between EV therapy and zinc depletion. Third, potential confounders such as baseline nutritional status, renal function, and cumulative prior therapies could not be fully excluded.

## Conclusion

Our findings suggest a possible association between zinc deficiency and EV-induced cutaneous adverse events, particularly SDRIFE-like rash. Zinc supplementation resulted in marked improvement of SDRIFE-like eruptions, whereas effects on other symptoms such as dry skin and dysgeusia were less pronounced.

Zinc supplementation may represent a simple, low-risk supportive measure that allows patients to continue EV therapy without additional dose modifications or interruptions. Prospective, larger-scale studies are warranted to validate these observations, clarify underlying mechanisms, and determine clinically meaningful thresholds for zinc screening and intervention during EV therapy, ultimately supporting treatment adherence and continuity.

## Data Availability

The original contributions presented in the study are included in the article/supplementary material. Further inquiries can be directed to the corresponding author.
